# GLP-1 Receptor Agonists in Fibromyalgia: Opioid Sparing Signals from Real World Data

**DOI:** 10.1192/j.eurpsy.2026.11477

**Published:** 2026-07-31

**Authors:** A. S. Verma, V. Sharma, A. Pathak, R. Gupta

**Affiliations:** 1 Psychiatry, Case Western Reserve University/ Metrohealth, Cleveland, United States; 2 Psychiatry, Dayanand Medical College & Hospital, Ludhiana, India; 3 Psychiatry, Cleveland Clinic Main Campus, Cleveland; 4 Psychiatry, Brigham & Women’s Faulkner Hospital; 5 Psychiatry, Harvard Medical School, Boston, United States

## Abstract

**Introduction:**

GLP-1 receptor agonists (GLP-1RAs) reduce appetite, energy intake, and serum glucose levels by activating glucagon-like peptide-1 (GLP-1) receptors. They, potentially, act on the central and peripheral nervous system to reduce nerve damage, inflammation, and pain hypersensitivity. Fibromyalgia (FM) presents with neuropathic pain and small fiber neuropathy, resulting in significant opioid use, estimates range from 20-55%. We hypothesize that GLP-1RAs might prove to be opioid sparing, reduce acute care use, and increase the overall mental health quality in FM

**Objectives:**

Compare the pain-related medication burden, acute-healthcare use, and psychiatric outcomes in adult patients with comorbid FM and obesity utilizing GLP-1RAs *vs.* other anti-obesity pharmacotherapies in a cohort design

**Methods:**

We conducted a retrospective, multi-network cohort study on the TriNetX Research Network. Adults (≥18 years) with an FM diagnosis (ICD-10-CM M79.7) and ≥12 months of data before and after the index drug initiation (GLP-1RAs) were eligible. The exposure was incident GLP-1RA initiation (semaglutide, liraglutide, exanatide, lixisenatide, dulaglutide, or tirzepatide) identified in EMR; the comparator was patients with FM without GLP-1RA exposure on a random outpatient visit. Propensity scores using demographics, BMI, type 2 DM, baseline pain score, PHQ-9/GAD-7 score, antidepressant/anxiolytic use, ED visits, and comorbidities (depression, anxiety, OSA, osteoarthritis). Primary outcome: Comparing any opioid fills. Secondary outcomes: Comparing neuromodulator/adjunct fills (duloxetine/ pregabalin/gabapentin/tizanidine), pain-related ED/hospital utilization, and symptom-burden proxies (PHQ-9 ≥10, GAD-7 ≥10, or pain NRS ≥4 where available). Analyses used measures of association, Kaplan-Meier, and number-of-instances. Patients with pre-window outcomes were excluded, and propensity score matching was applied (Figure 1).

**Results:**

After exclusions, opioid risk was lower with GLP-1RA vs comparator (risk 0.334 [7,574/22,686] vs 0.460 [13,690/29,767]; risk ratio 0.726, 95% CI 0.710-0.742). Opioid hazard was lower (HR 0.893, 95% CI 0.868-0.919; log-rank p<0.001). Opioid instances per patient were also lower (mean 1.34 vs 2.55; t=−22.35, p<0.001) (Figure 2). Neuromodulator/adjunct fills showed lower risk with GLP-1RA (0.246 vs 0.329; RR 0.748, 95% CI 0.732-0.765). A composite of pain-related ED/hospital outcomes favored GLP-1RA (risk 0.424 vs 0.494; RR 0.858, 95% CI 0.837-0.880) (Figure 3). Symptom-burden proxies were infrequent overall but nominally lower with GLP-1RA (RR 0.756, 95% CI 0.634-0.903).

**Image 1:**
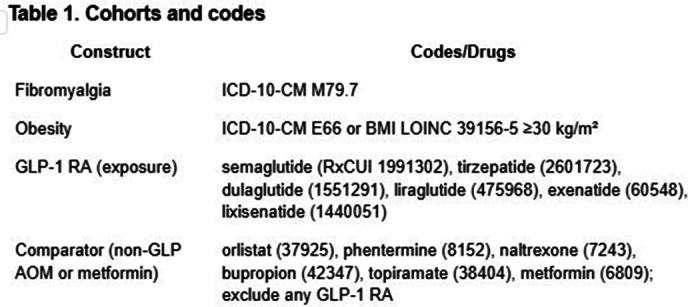
Image 1: Long description.

**Image 2:**
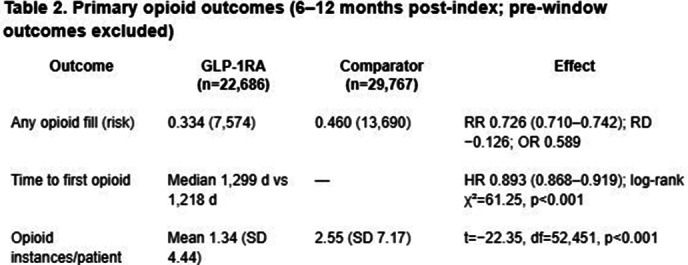
Image 2: Long description.

**Image 3:**
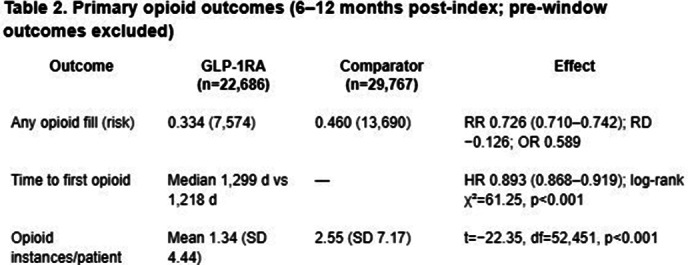
Image 3: Long description.

**Conclusions:**

In a large national EHR network, GLP-1RA initiation among adults with FM and obesity was associated with clinically meaningful reductions in opioid exposure and other pain-related utilization over 6-12 months. Our findings support prospective evaluation of GLP-1RAs as adjuncts for FM care and opioid-sparing strategies.

**Disclosure of Interest:**

None Declared

## Long descriptions

### Image 1: Long description

The table contains two columns: Construct and Codes/Drugs.

* Fibromyalgia: I C D-10-C M M 79.7.

* Obesity: I C D-10-C M E 66 or B M I L O I N C 39156-5 greater than or equal to 30 kg/m super 2.

* G L P-1 R A (exposure): semaglutide (R x C U I 1991302), tirzepatide (2601723), dulaglutide (1551291), liraglutide (475968), exenatide (60548), lixisenatide (1440051).

* Comparator (non-G L P A O M or metformin): orlistat (37925), phentermine (8152), naltrexone (7243), bupropion (42347), topiramate (38404), metformin (6809); exclude any G L P-1 R A.

Navigate back to Image 1:.

### Image 2: Long description

The table contains four columns: Outcome, G L P-1 R A (n=22,686), Comparator (n=29,767), and Effect.

* Row 1: Any opioid fill (risk). G L P-1 R A is 0.334 (7,574). Comparator is 0.460 (13,690). Effect shows R R 0.726 (0.710–0.742), R D minus 0.126, and O R 0.589.

* Row 2: Time to first opioid. G L P-1 R A is Median 1,299 d vs 1,218 d. Comparator is blank. Effect shows H R 0.893 (0.868–0.919) and log-rank chi-squared = 61.25, p < 0.001.

* Row 3: Opioid instances per patient. G L P-1 R A is Mean 1.34 (S D 4.44). Comparator is 2.55 (S D 7.17). Effect shows t = minus 22.35, d f = 52,451, p < 0.001.

Navigate back to Image 2:.

### Image 3: Long description

The table is titled Table 2. Primary opioid outcomes (6–12 months post-index; pre-window outcomes excluded). It contains four columns: Outcome, G L P-1 R A (n=22,686), Comparator (n=29,767), and Effect.

* Row 1: Any opioid fill (risk). G L P-1 R A is 0.334 (7,574). Comparator is 0.460 (13,690). Effect shows R R 0.726 (0.710–0.742), R D minus 0.126, and O R 0.589.

* Row 2: Time to first opioid. G L P-1 R A is Median 1,299 d vs 1,218 d. Comparator is blank. Effect shows H R 0.893 (0.868–0.919) and log-rank chi-squared equals 61.25, p less than 0.001.

* Row 3: Opioid instances/patient. G L P-1 R A is Mean 1.34 (S D 4.44). Comparator is 2.55 (S D 7.17). Effect shows t equals minus 22.35, d f equals 52,451, p less than 0.001.

Navigate back to Image 3:.

